# Polyoxometalate-based homochiral metal-organic frameworks for tandem asymmetric transformation of cyclic carbonates from olefins

**DOI:** 10.1038/ncomms10007

**Published:** 2015-12-18

**Authors:** Qiuxia Han, Bo Qi, Weimin Ren, Cheng He, Jingyang Niu, Chunying Duan

**Affiliations:** 1 State Key Laboratory of Fine Chemicals, Dalian University of Technology, Dalian 116024, China; 2 Key Laboratory of Polyoxometalate Chemistry of Henan Province, College of Chemistry and Chemical Engineering, Henan University, Kaifeng 475004, China; 3 Collaborative Innovation Center of Chemical Science and Engineering, Tianjin 300071, China

## Abstract

Currently, great interest is focused on developing auto-tandem catalytic reactions; a substrate is catalytically transferred through mechanistically distinct reactions without altering any reaction conditions. Here by incorporating a pyrrolidine moiety as a chiral organocatalyst and a polyoxometalate as an oxidation catalyst, a powerful approach is devised to achieve a tandem catalyst for the efficient conversion of CO_2_ into value-added enantiomerically pure cyclic carbonates. The multi-catalytic sites are orderly distributed and spatially matched in the framework. The captured CO_2_ molecules are synergistically fixed and activated by well-positioned pyrrolidine and amine groups, providing further compatibility with the terminal W=O activated epoxidation intermediate and driving the tandem catalytic process in a single workup stage and an asymmetric fashion. The structural simplicity of the building blocks and the use of inexpensive and readily available chemical reagents render this approach highly promising for the development of practical homochiral materials for CO_2_ conversion.

## Abstract (web summary)

Using a single catalyst to promote multiple distinct reactions without alteration in reaction conditions is an attractive synthetic goal. Here, to this end, the authors have developed a polyoxometalate-based metal-organic framework for the synthesis of cyclic carbonates from olefins.

## Graphical abstract

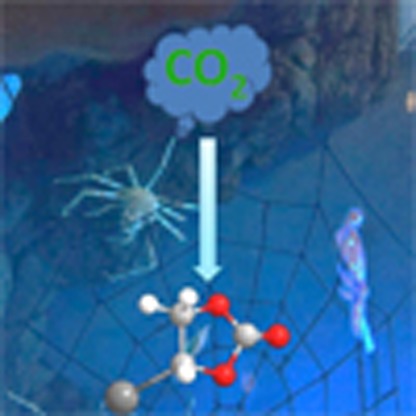

Recently, the selective and efficient conversion of light olefins with CO_2_ as a C_1_ building block into value-added enantiomerically pure cyclic carbonates has attracted great interest from chemists and governments due to its tremendous potential economic and environmental impact, especially considering the increasing worldwide greenhouse effect^1^. The general synthetic procedure includes two mechanistically distinct catalytic processes consisting of the asymmetric epoxidation of olefins, based on the research of Sharpless and others, and the asymmetric coupling epoxide with CO_2_ (refs 2, 3, 4, 5). Both of the catalytic processes require asymmetric (or associated) catalysts to ensure enantioselectivity, as well as a sequential separation and purification process of the products from the reagents, the catalysts and the associated materials. Because light olefins are the most inexpensive and readily available chemical building blocks, a one-pot procedure that processes an olefin through a chain of discrete catalytic steps with multiple transformations using a single workup stage is highly desirable for the environmentally benign conversion of carbon dioxide to fine chemicals^6^.

Inspired by natural enzymatic processes, chemists have attempted to emulate natural reaction cascades by developing auto-tandem catalytic reactions in which a substrate is catalytically transferred through two or more mechanistically distinct reactions without the addition of reactants or catalysts and without altering the reaction conditions^7^,^8^. Recently, homogeneous organo- and organometallic auto-tandem catalysts have been found to show high efficiency in solution^9^. This feature can be advantageous for separation and catalyst recovery by creating heterogenized catalysts using linkers to attach them to a support, such as high surface-area metal oxide materials, or alternatively, incorporating them within porous metal-organic frameworks (MOFs). One of the key advantageous of MOFs lies in their easily tunable composition in which the constituents' geometry, size and functionality can be widely varied^10^,^11^,^12^. Thus, an ongoing challenge for the development of auto-tandem catalytic processes has been beyond the selection and incorporation of two or more catalytic sites by modifying these moieties in the building block of the original MOFs, but includes the achievement of compatibility between the reaction intermediates and synergy of the multiple catalytic cycles^13^,^14^,^15^.

Polyoxometalates (POMs) are well-known catalysts studied in olefin epoxidation, with excellent thermal and oxidative stability towards oxygen donors^16^,^17^,^18^. By combining chiral organic catalysts and POM components in a single MOF (POMOF), these POMOF materials can initiate the asymmetric dihydroxylation of aryl olefins with excellent stereoselectivity^19^. Because MOFs have exhibited unrivalled efficiency as heterogeneous Lewis acid catalysts for the chemical conversion of CO_2_^20^,^21^,^22^,^23^, we envision that the combination of chiral organic catalysts, Lewis acid catalysts and oxidation catalysts would be a powerful approach to devise a tandem catalytic process for the chemical transformation of olefins to chiral cyclic carbonate.

By incorporating Keggin-type [ZnW_12_O_40_]^6−^ anions, zinc(II) ions, NH_2_-functionalized bridging links, NH_2_-bipyridine (NH_2_-**BPY**) and asymmetric organocatalytic groups, pyrrolidine-2-yl-imidazole (**PYI**) within one single MOF, herein we report the design and synthesis of two new enantiomorphs of POMOFs, ZnW−**PYI**1 and ZnW−**PYI**2, for the efficient conversion of CO_2_ into value-added enantiomerically pure cyclic carbonates. Because the intrinsic crystalline properties could provide precise knowledge about the nature and distribution of catalytically active sites and about the potential interactions between the catalytic sites and the adsorbed substrates, the use of POM-based homochiral MOFs offers the potential to facilitate analysis of the intermediates and the catalytic processes of each step of the tandem asymmetric transformation.

## Results

### Synthesis and characterization of the POMOFs

**Figure 1:**
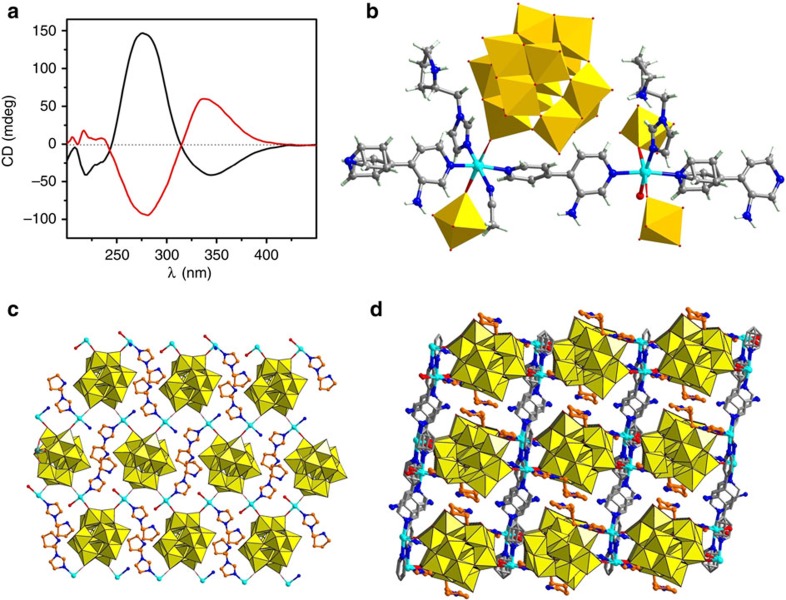
The structure and characterization of ZnW−PYIs. (**a**) Circular dichroism spectra of bulk crystals of ZnW−**PYI**1 (black) and ZnW−**PYI**2 (red), respectively. (**b**) Plot of the one-dimensional connections between the bipyridine ligands and the zinc centres, showing the coordination mode of the zinc centres. (**c**) Perspective view of the two-dimensional sheet connected by the polyoxometalate clusters and the zinc centres, the bipyridine ligands were omitted for clarity. (**d**) 3D open network of ZnW−**PYI**1 viewed down the *b* axis. H atoms and solvent molecules are omitted for clarity. Carbon, nitrogen and zinc are drawn in gray (orange for **PYI**), blue, and cyan, respectively, with ZnW_12_O_40_^ 6−^ shown as polyhedra.

The solvothermal reaction of TBA_4_W_10_O_32_, Zn(NO_3_)_2_·6H_2_O, NH_2_**-BPY** and L−N-*tert*-butoxy-carbonyl-2-(imidazole)-1-pyrrolidine (L−**BCIP)** in a mixed solvent of acetonitrile and H_2_O gave compound ZnW−**PYI**1 in a yield of 68% (Supplementary Fig. 1). Elemental analyses and powder X-ray diffraction (PXRD) indicated the pure phase of the bulk sample (Supplementary Fig. 2). Circular dichroism spectrum of the bulk sample of ZnW−**PYI**1 presented positive cotton effects at 276 nm (*θ*=146 mdeg) and negative cotton effects at 340 nm (*θ*=−41.2 mdeg). The entire spectrum was different from the spectra of the chiral adducts L−**BCIP** and L−**PYI** (Supplementary Fig. 3). ZnW−**PYI**2 was prepared similarly to ZnW−**PYI**1, except that D−**BCIP** replaced L−**BCIP**. The circular dichroism spectrum of ZnW−**PYI**2 exhibited the cotton effects that were opposite to ZnW−**PYI**1, as expected for a pair of enantiomers with a mirror–imager relationship (Fig. 1a). Single-crystal structural analysis revealed that ZnW−**PYI**1 and ZnW−**PYI**2 shared the same cell dimensions crystallized in the chiral space group *P*2_1_ (Supplementary Data 1 and 2, Supplementary Fig. 14–21).

### The crystal structures of POMOFs

**Figure 2:**
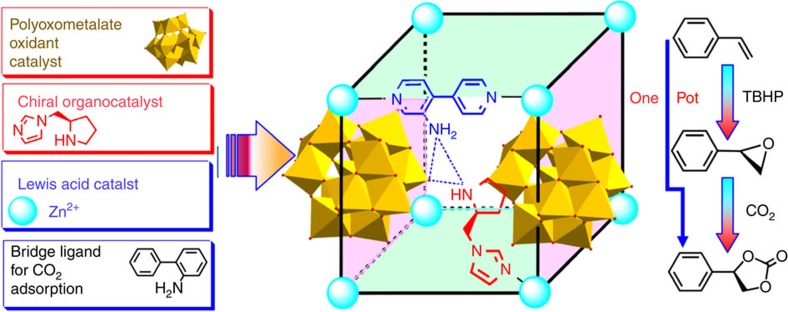
The design concept of achieving a tandem catalyst. Synthetic procedure of the metal-organic framework, showing the constitutive/constructional fragments of the MOF; and the schematic representation of tandem catalysis for the asymmetric cyclic carbonate transformation from olefins and carbon dioxide.

The asymmetric unit of ZnW−**PYI**1 consists of the Keggin anion ZnW_12_O_40_^6−^ and the cation [Zn_2_(NH_2_**-BPY**)_2_(H**PYI**)_2_(H_2_O)(CH_3_CN)]^6+^ (Supplementary Fig. 4). The precursor W_10_O_32_^4−^ anion is labile under hydrothermal conditions and favours to rearrangement into the more stable Keggin-type ZnW_12_O_40_^ 6−^ anion. Each of these ZnW_12_O_40_^ 6−^ anions connects to four zinc ions through the terminal oxygen atoms and acts as a four connector (Supplementary Fig. 5). Each of the two crystallographically independent zinc ions adopts a distorted octahedral geometry (Supplementary Fig. 6,7) and coordinates to two terminal oxygen atoms from different ZnW_12_O_40_^ 6−^ anions, forming a two-dimensional square grid sheet (Fig. 1b,c, Supplementary Fig. 8,9). Each of the NH_2_**-BPY** ligands coordinates to two different zinc ions and further bridges the sheets to form a three-dimensional framework with one-dimensional chiral channels along the *b* axis (Fig. 1d, Supplementary Fig. 10,11). The accessible empty space of the framework is approximately 289.1 Å^3^ (8.1%), as calculated from PLATON analysis, suggesting the possibility that the MOF-based material ZnW−**PYI1** can absorb substrates within its pores^24^. Chiral **PYI** was located in the pores of the MOF, with the butoxycarbonyl of L−**BCIP** removed simultaneously in the reaction and the pyrrolidine nitrogen was protonated. The unique redox properties of ZnW_12_O_40_^ 6−^, with its oxygen-enriched surface, provide sufficient driving force for the transformation of the catalytic precursors to the active intermediate of the epoxidation^25^,^26^. The chiral **PYI**s act as cooperative catalytic sites to forge a crucial reaction centre that enhances the activities of the oxidants and drives the catalysis asymmetrically^27^,^28^. Detailed structural analyses reveal that one zinc ion has a water molecule at its sixth coordination position, while the other is associated with an acetonitrile molecule. The removability of the coordinated water molecules indicates the potential of ZnW−**PYI**1 to act as an active Lewis acid catalyst (Supplementary Fig. 12). These amine groups in NH_2_-**BPY** are positioned around the inner surface of the channels and act as accessible sites to activate CO_2_ directly, enabling the catalytic performance in the coupling of carbon dioxide with epoxide under relatively mild conditions (Fig. 2)^29^,^30^,^31^,^32^.

### Separated tandem catalysis

**Figure 3:**
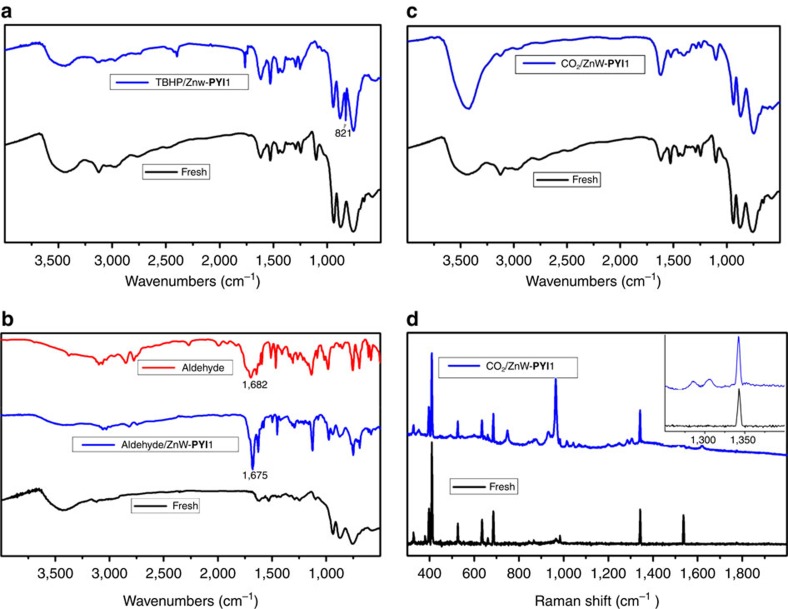
The spectroscopic studies of ZnW−PYI1. (**a**) Infrared (IR) spectra of fresh and TBHP-oxidized ZnW−**PYI**1. (**b**) IR spectra of fresh and cinnamaldehyde-incorporated ZnW−**PYI**1. (**c**,**d**) IR spectra and Raman spectra of fresh (dark line) and CO_2_-adsorbed ZnW−**PYI**1 (blue line), respectively.

The transformation of asymmetric epoxidation was initially examined using styrene and *t*-butylhydroperoxide (TBHP, 70% in decane) as the oxidant, along with ZnW−**PYI**1 (0.1% mol ratio) in a heterogeneous reaction at 50 °C for 5 days. As shown in Table 1 (entries 1–4), the result revealed the successful execution of our POMOF design, showing excellent yield (92%) and enantioselectivity (79% enantiomer excess (ee)) for (*R*)-styrene oxide (Supplementary Fig. 22, Supplementary Table 6). The control experiments showed that L−**PYI** and its hydrochloride salt could not initiate the reaction under similar conditions. The use of L−**PYI** and Zn_3_[ZnW_12_O_40_] as homogeneous catalysts gave a conversion of 55% and an ee value of 18%. The higher conversion in the case of the ZnW−**PYI**1 system was attributed to the suitable distribution of pairs of the chiral **PYI** moiety and the ZnW_12_O_40_^ 6−^ oxidant catalyst. Infrared of the catalyst impregnated with TBHP revealed a new *v*(O–O) band at 821 cm^−1^, which indicated that the active intermediate peroxotungstate was formed during the epoxidation process (Fig. 3a)^33^. From a mechanistic perspective, the formation of hydrogen bonds between the pyrrolidine N atom and the terminal oxygen atoms of the ZnW_12_O_40_^ 6−^ with the interatomic separation N(12)···O(7), approximately 2.8 Å, initially activated the corresponding W=O_t_ (O_t_=terminal oxygen) and generated an active peroxide tungstate intermediate, ensuring the smooth progress of the reaction (Supplementary Table 2–5). These hydrogen bonds then enforced the proximity between the conventional electrophilic oxidant and the chiral directors to provide additional steric orientation, driving the catalysis to proceed in a stereoselective manner^19^,^34^.

Solids of ZnW−**PYI**2 exhibited similar catalytic activities but gave products with the opposite chirality in the asymmetric epoxidation of styrene. The use of such a catalyst can be extended to other substituted styrene substrates and *α, β*-unsaturated aldehydes with comparable activity and asymmetric selectivity. The presence of a C=O stretching vibration at 1,675 cm^−1^ (cf. 1682, cm^−1^of the free aldehyde) in the infrared spectra of the catalyst impregnated with a CH_2_Cl_2_ solution of cinnamaldehyde suggested the absorbance and activation of cinnamaldehyde in the cavities of ZnW−**PYI**1 (Fig. 3b).

The transformation of the asymmetric coupling of CO_2_ to styrene oxide was examined using the racemic styrene oxide and CO_2_ in free solvent, along with ZnW−**PYI**1 (0.1% mol ratio) and cocatalyst TBABr (1% mol ratio), in a heterogeneous mixture at 50 °C and 0.5 MPa for 48 h, as shown in Table 1 (entry 5). The result exhibited excellent reaction efficiency (>99% yield) for phenyl(ethylene carbonate) (Supplementary Table 7). The control experiments demonstrated that no detectable conversion was observed for the model reaction in the absence of ZnW−**PYI**1 or the ammonium salt cocatalyst. It is postulated that the directed coordination of the ZnW_12_O_40_^ 6−^ anion to the zinc ions results in significant activation of the MOF on the acid surface, facilitating the coupling step because of its additional labile ligand sites, possibly through the provision of a carbon dioxide. The surface properties of the nanocation also meet a number of criteria for the carbon dioxide coupling reaction, such as an overall positive charge, the presence of coordinated water and the presence of functional groups for acid catalysis. Enantiospecific phenyl(ethylene carbonate) could be obtained through the coupling of CO_2_ to the (*R*) or (*S*)-styrene oxide, catalysed with excellent reaction efficiency (>99% yield) and high enantioselectivity (>90% ee) (Supplementary Fig. 23, Supplementary Table 8) by ZnW−**PYI**s (Table 1, entries 6,7). The retention of chirality through the asymmetric coupling process demonstrated that selective ring opening occurred preferentially at the methylene C−O bond of the terminal epoxides^5^,^32^. The high selectivity of the asymmetric transformation reflected that CO_2_ molecules were also activated, providing favourable conditions for a fast reaction with epoxide by nucleophilic attack, avoiding the racemization of the epoxide through premature ring opening. It is postulated that these NH_2_ molecules in channels not only enhanced the reaction rate by increasing the concentration of CO_2_ substrate around its reactive centre but also increased the electron cloud density of activated CO_2_, enabling the cyclic carbonate ring formation^35^. In contrast to Ahn's group reported that combination of Lewis acid and base give highly active for the CO_2_ cycloaddition to epoxide because of cooperative activation of epoxide by acid-base^22^, the Lewis acid catalytic zinc centres restricted within the ZnW-**PYI**s channels have the potential to interact synergistically with CO_2_ in view of spatial location matching of catalytic sites in MOFs, such that an intramolecularly cooperative catalysis is proposed to contribute to the high activity and excellent stereochemical control of the given reactions^36^.

The irreversible CO_2_-adsorption and desorption isotherms of ZnW−**PYI**1 may be attributed to the chemical adsorption onto the amine of NH_2_**-BPY** (Supplementary Fig. 13) (ref. 37). The infrared spectrum of activated ZnW−**PYI**1 in a vacuum and after the introduction of 1 bar of CO_2_ at room temperature clearly shows the loss of the NH stretching vibration peak at 3,126 cm^−1^, confirming that the CO_2_ was adsorbed and activated by NH_2_ groups in the channels of ZnW−**PYI**1 (Fig. 3c) (refs 38, 39). The Raman spectra of the MOF in a vacuum and after the introduction of 1 bar of CO_2_ showed peaks at 1,285 cm^−1^ (ref. 40), further supporting that CO_2_ molecules were adsorbed within the MOFs (Fig. 3d). Mechanistically, this reaction is based on a nucleophilic cocatalyst that activates the epoxide to form an alkoxide. This intermediate can then react with activated carbon dioxide to ultimately yield the cyclic carbonate.

### One-pot asymmetric catalysis

Because chiral ZnW−**PYI**s demonstrated their ability as effective catalysts in the epoxidation of styrene and the coupling reaction, the one-pot synthesis was applicable to a range of different substrates. As shown in Table 1 (entries 9–11), by heating a reaction mixture of styrene, TBHP and CO_2_ with catalyst ZnW−**PYI**1 to 50 °C, the asymmetric epoxidation and the CO_2_ asymmetric coupling could be smoothly completed with a single workup stage. The target (*R*)-phenyl(ethylene carbonate) was obtained in 92% yield with 80% ee (Supplementary Fig. 24, Supplementary Table 9). The removal of ZnW−**PYI**1 by filtration after 48 h stopped the reaction, and the filtrate afforded nearly no additional conversion after stirring for another 48 h. These observations suggest that ZnW−**PYI**1 is a true heterogeneous catalyst. ZnW−**PYI**1 solids could be isolated from the reaction suspension by simple filtration. The catalysts could be reused at least three times with moderate loss of activity (from 92 to 88% yield) and with a slight decrease in selectivity (from 80 to 77% ee) (Supplementary Table 10). The index of PXRD patterns of the ZnW−**PYI**1 bulky sample filtered from the catalytic reaction revealed that the crystallinity was maintained. The ZnW−**PYI**2 solids exhibited similar catalytic activities but gave products with opposite chiralities in the asymmetric auto-tandem epoxidation/coupling of styrene.

## Discussion

**Figure 4:**
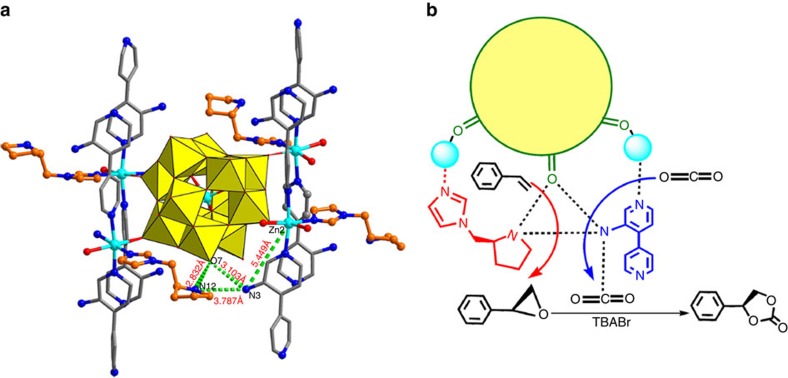
The spatial relationship of multiple catalytic sites and synergetic catalysis. (**a**) Schematic diagram of the catalytic site distribution in ZnW−**PYI**1, showing the interactions of the catalytic sites. (**b**) Diagram of potential mechanism for the auto-tandem catalysis.

It was found that performing the reaction through a one-pot process could greatly shorten the reaction time to 4 days, and the configuration was maintained throughout both of the steps. It is clear from the crystal structures of the ZnW−**PYI**s that the smooth and stereoselective conversion of the olefins into the epoxide can be attributed to the hydrogen bonding interaction between the spatially matched organocatalyst **PYI** and the oxidation catalyst ZnW_12_O_40_^ 6−^ and to the potential π–π interaction between the benzene ring of styrene oxide and the imidazole ring of **PYI**. Because the CO_2_ molecules adsorbed in the channels of ZnW−**PYI**1 are activated by NH_2_ groups, the weak hydrogen bonding interactions between the chiral amine group N(12) and the amino group N(3) (interatomic separation of 3.79 Å) is believed to enforce the proximity between the activated CO_2_ and the terminal epoxide (Fig. 4). The well-matched positions and the suitable interactions provide a promising route for nucleophilic attack at the methylene C−O bond, ensuring chiral retention in the coupling process. The Lewis acid catalytic zinc centres restricted within the MOF channels have the potential to interact synergistically with CO_2_ such that the compatibility between the reaction intermediates and the synergy of the multiple catalytic cycles allow the auto-tandem reaction to proceed smoothly and efficiently^36^. Most importantly, the multi-catalytic sites with orderly distribution and spatial matching in the three-dimensional open framework provide favourable conditions for the auto-tandem reaction and avoid cross-interference.

The use of this catalyst can be extended to other styrene derivatives with comparable activity and asymmetric selectivity. In contrast to the smooth reaction of substrates 9–11, the one-pot catalytic reaction in the presence of cinnamaldehyde only gave 25% conversion under the same reaction conditions (Table 1, entry 12). It is suggested that the aldehyde group has the potential to interact with the NH_2_ group, inactivating these sites for the activation and concentration of CO_2_, which further substantiates the pivotal role of NH_2_ in the one-pot process.

We have developed an asymmetric auto-tandem epoxidation/cycloaddition catalytic reaction catalysed solely by chiral POMOFs, which proceeds in a highly enantioselective manner for the efficient conversion of light olefins into value-added enantiomerically pure cyclic carbonates in a one-pot procedure. The results demonstrated that asymmetric auto-tandem catalysis is an atom-economical and environmentally benign synthetic method for producing useful chiral compounds.

Future work will focus on design structurally diverse chiral POMOFs and optimizing such reactions. The structural simplicity of catalyst and the use of inexpensive and readily available chemical reagents render this approach highly promising for the development of practical homochiral materials for asymmetric catalytic reactions.

## Methods

### Reagents and Syntheses

All chemicals were of reagent grade quality obtained from commercial sources and used without further purification. All epoxides were purchased from Acros and distilled under a nitrogen atmosphere from CaH_2_ prior to use. Carbon dioxide (99.995%) was purchased from Dalian Institute of Special Gases and used as received. All manipulations involving air- and/or water-sensitive compounds were carried out in glove box or under dry nitrogen using standard Schlenk techniques. L− and D−N-*tert*-butoxycarbonyl-2-(imidazole)-1-pyrrolidine (L− or D−**BCIP**) (ref. 41), 3-amino-4,4′-bipyridine (NH_2_-**BPY**) (ref. 42), and [(n-C_4_H_9_)_4_N]_4_ [W_10_O_32_] (ref. 43) were prepared according to the literature methods.

### Elemental analyses

Elemental analyses of C, H and N were performed on a Vario EL III elemental analyser.

### Inductively coupled plasma

W and Zn analyses were performed on a Jarrel-AshJ-A1100 spectrometer.

### Fourier translation infrared spectrum (FT-IR)

FT-IR spectra were recorded as KBr pelletson JASCO FT/IR-430.

### Powder X-ray diffractograms

PXRDs were obtained on a Riguku D/Max-2,400 X-ray diffractometer with Cu sealed tube (*λ*=1.54178 Å).

### Circular dichroism spectrum

Circular dichroism spectra were measured on JASCO J-810 with solid KBr tabletting.

### Laser-Raman spectrum

Raman spectroscopy (Lab Raman HR Evolution) measurements were performed using a solid state 785 nm laser. A laser power of 1–2.5% was used to avoid degradation of the sample under the laser beam during the Raman measurements.

### Thermogravimetric analysis

Thermogravimetric analyses were performed on a Mettler-Toledo TGA/SDTA851 instrument and recorded under N_2_ or under air, upon 14 equilibration at 100 °C, followed by a ramp of 5 °C min^−1^ up to 800 °C.

### Gas adsorption isotherms

Gas adsorption isotherms were collected using a Micromeritics 3Hex 128 instrument. As-synthesized crystals were thoroughly washed with anhydrous dichloromethane and dried under argon flow, approximately 100 mg of each sample was added into a pre-weighed sample analysis tube. The samples were degassed at 100 °C under vacuum for 24–48 h until the pressure change rate was no more than 3.5 mTorr min^−1^. Ultra high purity (UHP) grade N_2_ and CO_2_ gas adsorbates (99.999 %) were used in this study.

### Nuclear magnetic resonance

^1^H and ^13^C nuclear magnetic resonance (NMR) spectra were recorded on a Varian INOVA-400 MHz type (^1^H, 400 MHz; ^13^C, 400 MHz) spectrometer. Their peak frequencies were referenced versus an internal standard (tetramethylsilane (TMS)) shifts at 0 p.p.m. for ^1^H NMR and against the solvent, chloroform-D at 77.0 p.p.m. for ^13^C NMR, respectively.

### High-performance liquid chromatography

High-performance liquid chromatography analysis was performed on Agilent 1,150 using a ChiralPAk OD−H column or AD−H column purchased from Daicel Chemical Industries, Ltd.

### Crystallography

Data of POMOFs ZnW−**PYI**s were collected on a Bruker SMART APEX CCD diffractometer with graphite-monochromated Mo-Kα (*λ*=0.71073 Å) using the SMART and SAINT programs^44^,^45^. Routine Lorentz polarization and Multi-scan absorption correction were applied to intensity data. Their structures were determined and the heavy atoms were found by direct methods using the SHELXTL-97 program package^46^. The remaining atoms were found from successive full-matrix least-squares refinements on *F*^2^ and Fourier syntheses. Not all the non-hydrogen atoms were refined anisotropically. Hydrogen atoms within the ligand backbones were fixed geometrically at their positions and allowed to ride on the parent atoms. Crystallographic data for ZnW−**PYI**s are summarized in Supplementary Table 1.

### Synthesis of ZnW−**PYI**1

A mixture of [(n-C_4_H_9_)_4_N]_4_[W_10_O_32_] (66.4 mg, 0.02 mmol), Zn(NO_3_)_2_·6H_2_O (60.0 mg, 0.20 mmol), NH_2_-**BPY** (10.3 mg, 0.06 mmol) and L−**BCIP** (7.5 mg, 0.03 mmol) in mixed water (4.0 ml) and acetonitrile (2.0 ml) was stirred and its pH value was adjusted to 4.3 with 1 mol l^−1^ acetic acid (HAc). The resulting suspension was sealed in a 25 ml Teflon-lined reactor and kept at 130 °C for 3 days. After cooling the autoclave to room temperature, yellow rod-like single crystals were separated, washed with water and air-dried. (Yield: calculated (calcd) 68% based on [(n-C_4_H_9_)_4_N]_4_[W_10_O_32_]). Elemental analyses and inductively coupled plasma calcd (%) for C_40_H_54_N_14_O_41_W_12_Zn_3_: C 12.68, H 1.44, N 5.18, Zn5.18, W 58.22; Found: C 12.64, H 1.41, N 5.20, Zn5.22, W 58.24 for ZnW−**PYI1**. IR (KBr): 3,440 (s), 3,123(w), 1,619(s), 1,532(s), 1,247 (s), 1,103(w), 938(s), 872(s), 756(versus) per cm.

### Synthesis of ZnW−**PYI**2

The preparation of ZnW−**PYI**2 was similar to that of ZnW−**PYI**1, except that D−**BCIP** (50.0 mg, 0.2 mmol) replaced L−**BCIP**. (Yield: *ca.* 68% based on [(n-C_4_H_9_)_4_N]_4_[W_10_O_32_]). Elemental analyses and inductively coupled plasma calcd (%) for C_40_H_54_N_14_O_41_W_12_Zn_3_: C 12.68, H 1.44, N 5.18, Zn5.18, W 58.22; Found: C 12.64, H 1.42, N 5.17, Zn 5.20, W 58.25 for ZnW−**PYI2**. IR (KBr): 3,443 (s), 3,124(w), 1,618(s), 1,531(s), 1,248 (s), 1,104(w), 939(s), 873(s), 757(versus) per cm.

### Typical one-pot procedure for asymmetric catalysis

Catalyst (0.01 mmol), TBABr (0.1 mmol), TBHP (20 mmol) and styrene (10 mmol) was added to a Schlenk flask (50 ml) equipped with a three-way stopcock. Then CO_2_ was charged into the autoclave, and the pressure (0.5 MPa) was kept constant during the reaction. The autoclave was put into a bath and heated to the 50 °C. After the expiration of the desired time, the excess gases were vented. The remaining mixture was degassed and fractionally distilled under reduced pressure or purified by column chromatography on silica gel to obtain the cyclic carbonate.

## Additional information

**Accession codes:** The X-ray crystallographic coordinates for structures reported in this study have been deposited at the Cambridge Crystallographic Data Centre (CCDC), under deposition numbers 1063826–1063827. These data can be obtained free of charge from The Cambridge Crystallographic Data Centre via www.ccdc.cam.ac.uk/data_request/cif.

**How to cite this article:** Han, Q. *et al.* Polyoxometalate-based homochiral metal-organic frameworks for tandem asymmetric transformation of cyclic carbonates from olefins. *Nat. Commun.* 6:10007 doi: 10.1038/ncomms10007 (2015).

## Supplementary Material

Supplementary Information Supplementary Figures 1-24, Supplementary Tables 1-10, Supplementary Methods and Supplementary References

Supplementary Data 1 Crystal data of complex Zn-PYI1-L

Supplementary Data 2 Crystal data of complex Zn-PYI2-D

## Figures and Tables

**Table 1:**
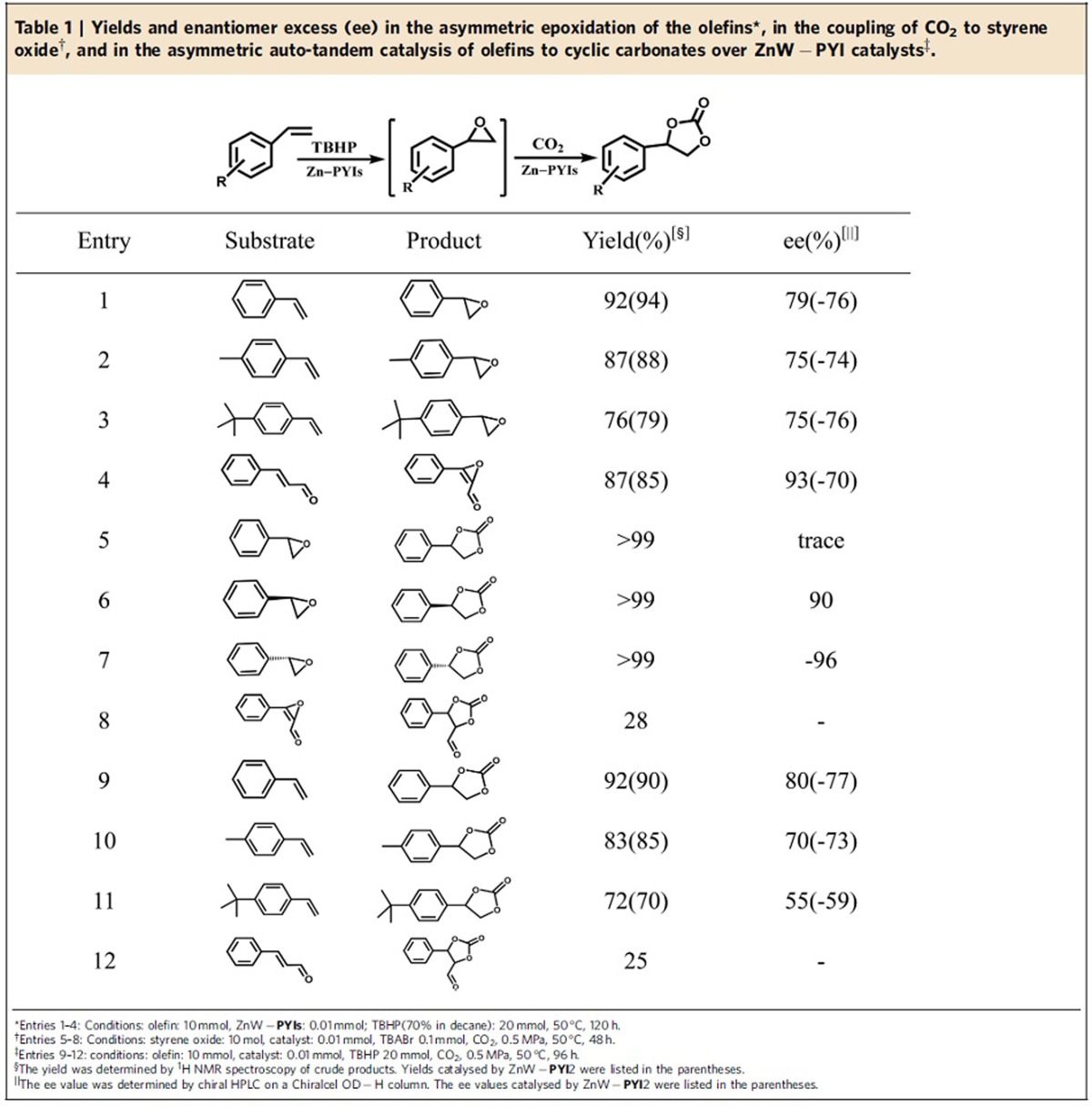
Yields and enantiomer excess (ee) in the asymmetric epoxidation of the olefins*, in the coupling of CO_2_ to styrene oxide^†^, and in the asymmetric auto-tandem catalysis of olefins to cyclic carbonates over ZnW−**PYI** catalysts^‡^.
