# Supplementary material for: Polyoxometalate-based homochiral metal-organic frameworks for tandem asymmetric transformation of cyclic carbonates from olefins
Source: Nat Commun. 2015 Dec 18;6:10007. doi: 10.1038/ncomms10007 (PMC4703842; doi:10.1038/ncomms10007)
Supplement: Supplementary Information — Supplementary Figures 1-24, Supplementary Tables 1-10, Supplementary Methods and Supplementary References [file ncomms10007-s1.pdf]

Supplementary Figures

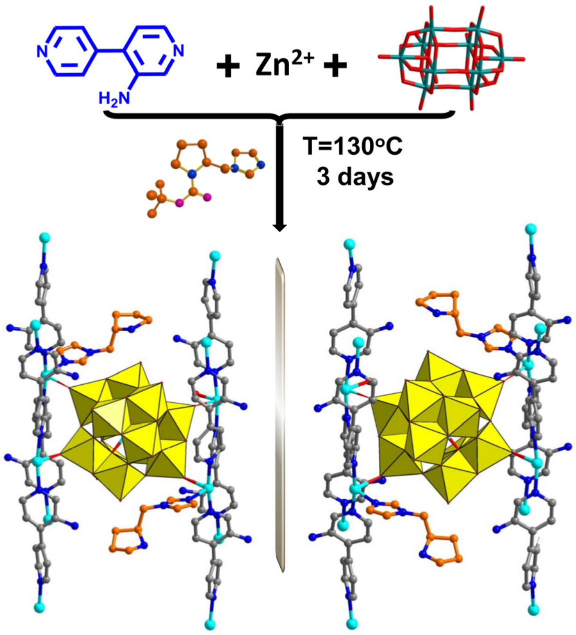

Supplementary Figure 1. Perspective view of the synthetic procedure and representation of the mirror image structures of ZnW−**PYI**1 (left) and ZnW−**PYI**2 (right). Showing the rearrangement of precursor $[W_{10}O_{32}]^{4-}$.

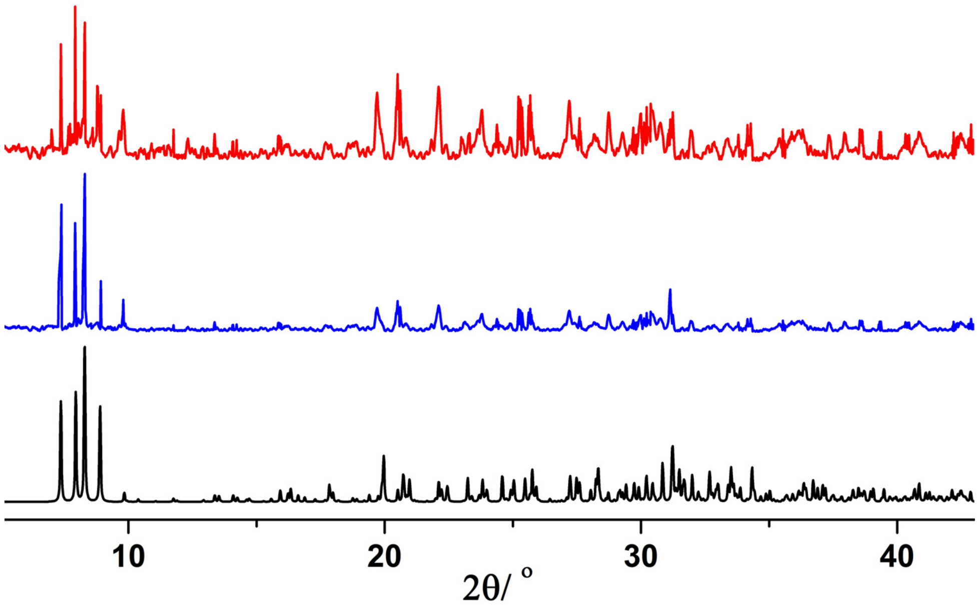

Supplementary Figure 2. PXRD pattern of ZnW−PYI1. its calculated pattern based on the single-crystal solution and after three runs of the tandem reaction (bottom-Simulated, middle-Experimental, top-Recovery catalyst after three runs). Showing that four strong peaks (101), (101), (011) and (110) were maintained during the catalytic processes.

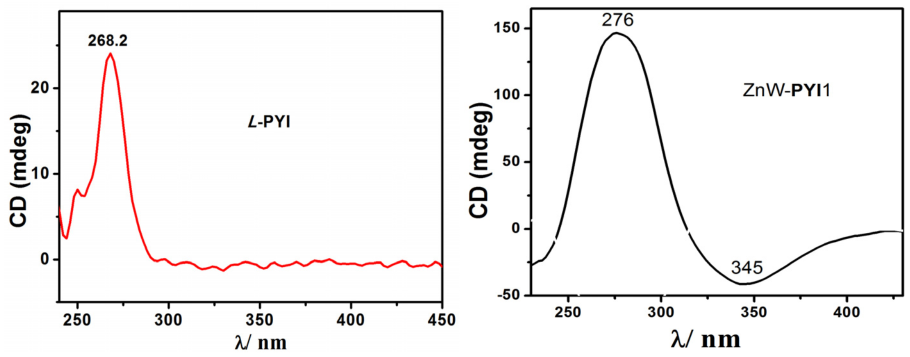

Supplementary Figure 3. CD spectra of bulk crystals of L−PYI (left) and ZnW−PYI1 (right). The profile of the CD spectrum of ZnW−**PYI**1 is totally different from that of its precursor, *L*−**PYI**, which only shows a single positive Cotton effect at 268 nm. In the long-wavelength spectral region where ZnW−**PYI**1 is CD-active (above 345 nm; vertical dashed line in the inset), *L*−**PYI** is almost CD silent. In contrast, ZnW−**PYI**1 exhibits strong Cotton effects up to 345 nm, the spectral region where the characteristic oxygen-to-tungsten charge-transfer bands of polyanions occur. Thus the chiral *L*−**PYI** introduced into the POMOF facilitate transfer of chirality from *L*−**PYI** to the POM, and the induced optical activity in ZnW−**PYI**1 is quite distinct.

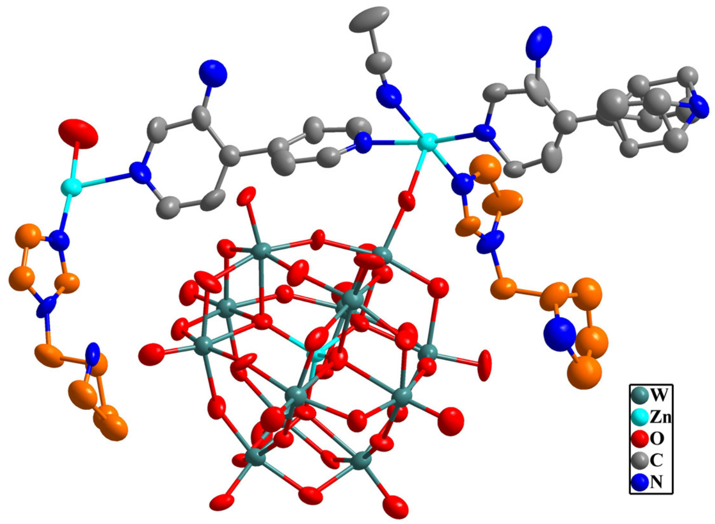

Supplementary Figure 4. Ellipsoid diagram of ZnW−PYI1 in an asymmetric unit with selected labelling scheme. Free CH_3CN molecule and all H atoms are omitted for clarity. The asymmetric unit of ZnW−**PYI**1 consists of the Keggin anion $ZnW_{12}O_{40}^{6-}$, the cation $[Zn_2(NH_2$-**BPY**$)_2$ $(H$**PYI**$)_2(H_2O)(CH_3CN)]^{6+}$ and one free CH_3CN molecule.

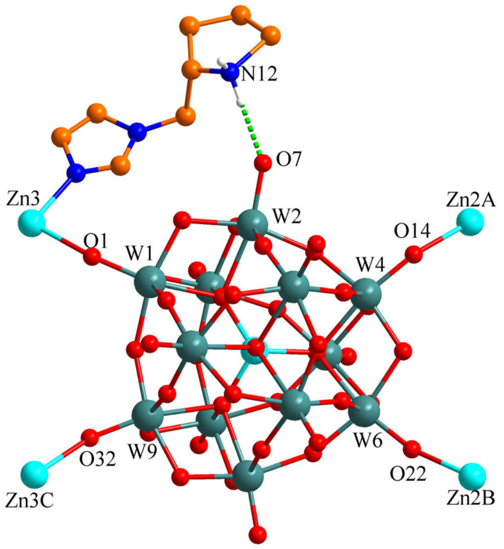

Supplementary Figure 5. Ball-and-stick representation of the coordination environments of $ZnW_{12}O_{40}^{6-}$ with selected labelling scheme, displaying hydrogen-bonding interactions between protonated pyrrolidine rings and the $ZnW_{12}O_{40}^{6-}$ in ZnW−**PYI**1. (Symmetry code: A *x*-1, *y*, *z*; B -*x*, *y*-1/2, -*z*+1; C -*x*, *y*-1/2, -*z*)

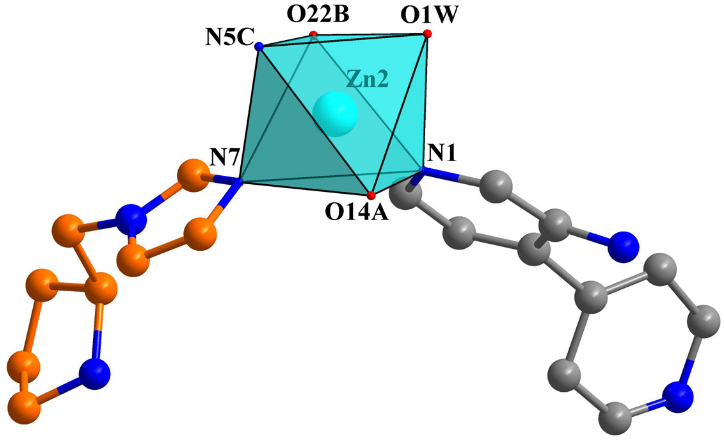

Supplementary Figure 6. Polyhedron of representation of the coordination environments of Zn(2) ion with selected labelling scheme in ZnW−**PYI**1(Symmetry code: A *x*+1, *y*, *z*; B -*x*, *y*+1/2, -*z*+1; C *x*+1, *y*, *z*+1). The crystallographically independent Zn(2) ion in ZnW−**PYI**1 adopts a distorted octahedral geometry. In $[Zn(2)(H_2O)(O_t)_2(N)_3]^{2+}$, Zn(2) atom is defined by one oxygen atom from coordinated water [Zn(2)–O(1W): 2.205(14)Å], two terminal oxygen atoms of $ZnW_{12}O_{40}^{6-}$ [Zn(2)–O(14A) 2.125(9) Å, Zn(2)–O(22**B**) 2.158(7) Å], two nitrogen atoms from NH_2-**BPY** [Zn(2)–N(1) 2.121(10) Å, Zn(2)–N(5C) 2.218(10) Å] and one nitrogen atom from **PYI**[Zn(2)–N(7) 2.088(11) Å].

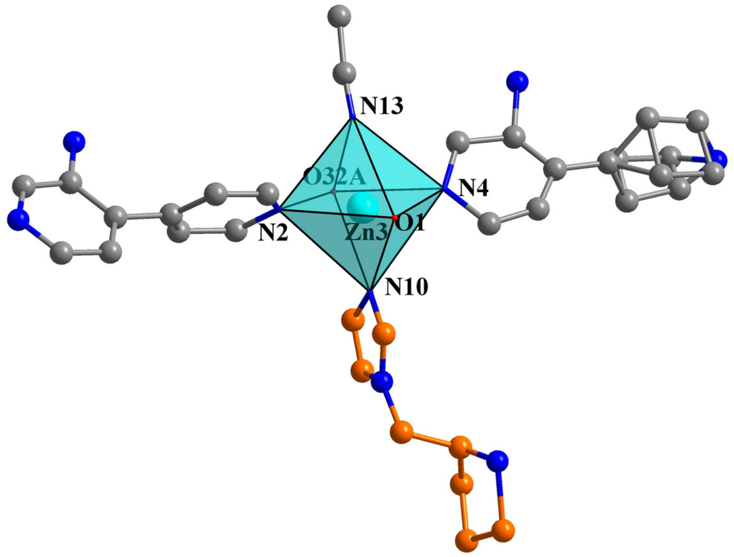

Supplementary Figure 7. Polyhedron of representation of the coordination environments of Zn(3) ion with selected labelling scheme in ZnW−**PYI**1 (Symmetry code**:** A -*x*, *y*+1/2, -*z*). The crystallographically independent Zn(3) ion in ZnW−**PYI**1 adopts a distorted octahedral geometry. In $[Zn(3)(O_t)_2(N)_4]^{2+}$, Zn(3) atom is defined by two terminal oxygen atoms of $ZnW_{12}O_{40}{}^{6-}$ [Zn(3)–O(1) 2.220(9) Å, Zn(3)–O(32A) 2.168(8) Å], two nitrogen atoms from NH_2-**BPY** [Zn(3)–N(2) 2.134(9) Å, Zn(3)–N(4) 2.094(9) Å], one nitrogen atom from **PYI** [Zn(3)–N(10) 2.075(11) Å], and one nitrogen atom from CH_3CN [Zn(3)–N(13) 2.255(14) Å].

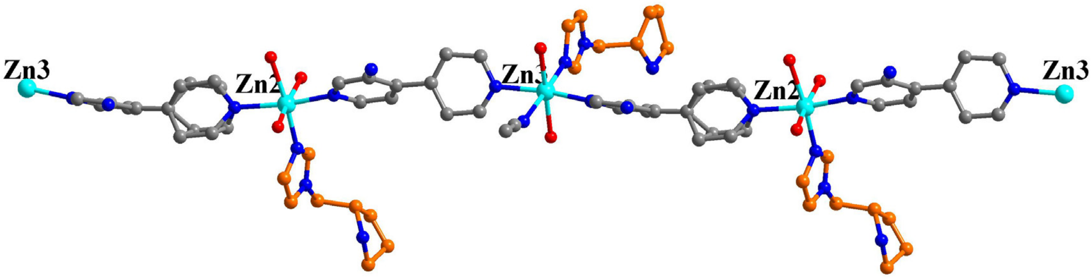

Supplementary Figure 8. The 1D chain of ZnW−**PYI**1 produced by two crystallographically independent Zn(II) ions linking with NH_2-**BPY** alternatively.

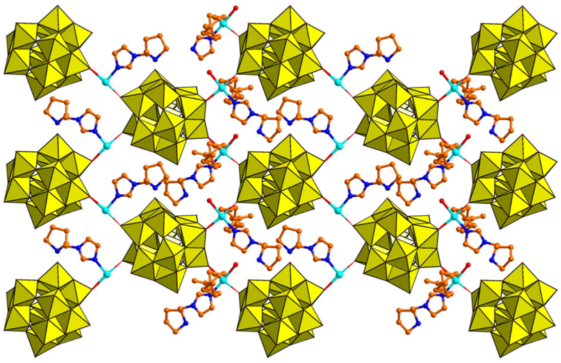

Supplementary Figure 9. The 2D network of ZnW−PYI1 showing the coordination environment of POM. Each of these $ZnW_{12}O_{40}^{6-}$ anions connectes to four zinc ions through the terminal oxygen atoms and acts as a four connector. Each of the two crystallographically independent zinc ions coordinates to two terminal oxygen atoms from different $ZnW_{12}O_{40}^{6-}$ anions, forming a 2D square grid sheet. These coordinated **PYI** molecules located above or beneath the 2D layer with the butoxycarbonyl of *L*−**BCIP** removed in the reaction simultaneously and the pyrrolidine N atoms were protonated, ensuring the chirality of the whole layer. Hydrogen bonds were found between the protonated pyrrolidine N atoms and the $ZnW_{12}O_{40}^{6-}$ anion.

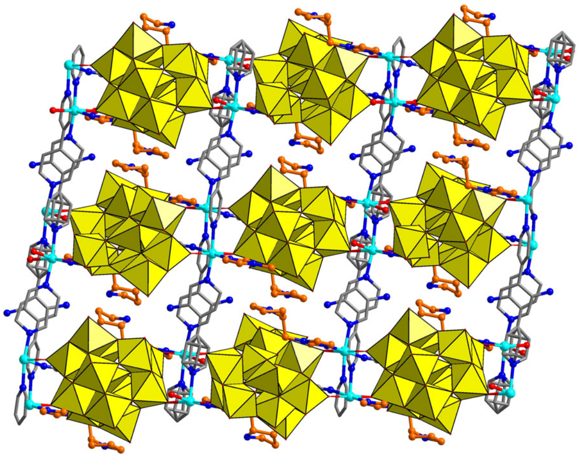

Supplementary Figure 10. Perspective view of the 3D open network of ZnW−PYI1 viewed down the *b*-axis. Carbon, nitrogen and zinc are drawn in gray (orange for **PYI**), blue, and cyan, respectively, with $ZnW_{12}O_{40}^{6-}$ shown as polyhedra. H-atoms and solvent are omitted for clarity. As seen in Figure S10, the 2D sheets were further stacked by bridging ligand NH_2-BPY linking with Zn(2) and Zn(3) in an alternative way to produce a 3D infinite structure with 1D channels along the *b* axis. The porous structure not only ensures the highly regular 3D porosity for the reaction substrates ingress and egress through these channels, but also promotes the favored interaction between amine groups and CO_2 molecules.

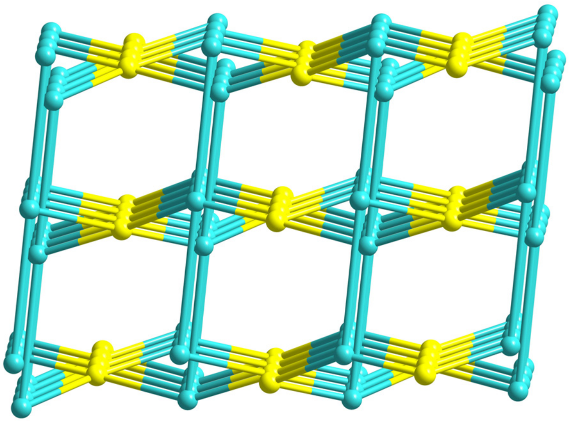

Supplementary Figure 11. A schematic representation of the 3D *nbo* topology ($6^4 8^2$) of ZnW−PYI1. (aqua ball for Zn^{2+} ion, yellow ball for $ZnW_{12}O_{40}^{6-}$, respectively). Both Zn^{2+} ion and $ZnW_{12}O_{40}^{6-}$ can be viewed as 4-connected nodes. Thus, topologically, the framework can be classified as a 3D (4,4)-connected ***nbo*** net with the point symbol ($6^4 8^2$).

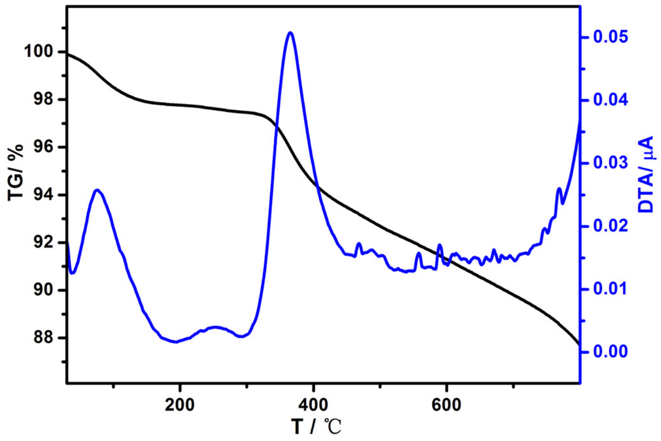

Supplementary Figure 12. TG-DTA curve of ZnW−PYI1. Thermogravimetric analysis (TG-DTA) was performed in flowing air atmosphere with a heating rate of 10 °C min^{-1} on the pure sample for ZnW−**PYI**1. The TG curve of ZnW−**PYI**1 exhibits two steps of weight loss processes. The first weight loss of 2.67 % (cacld. 2.64 %) between 18 and 310 °C corresponds to the loss of one coordinated water molecule and two CH_3CN molecules. The second weight loss of 7.90 % up to 720 °C is assigned to the removal of two NH_2-**BPY** and two H**PYI** en ligands. In the corresponding DTA curve, the two strong exothermal peaks at 76 °C and 364 °C arised from the combustion of organic ligands together with the collapse of the framwork of ZnW−**PYI**1. ZnW−**PYI**1 exhibits high chemical and thermal stability and meets most of the prerequisites as an ideal platform for heterogeneous catalysis.

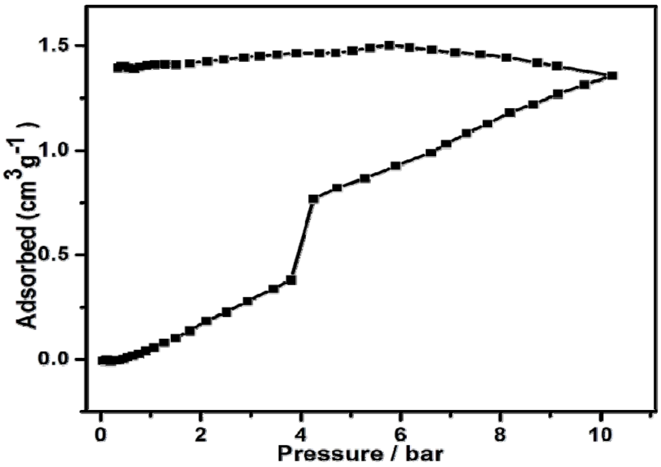

Supplementary Figure 13. CO_2-adsorption and desorption isotherms at 293K for ZnW−PYI1. To demonstrate the potential of the ZnW−**PYI**1 in CO_2 gas absorption, we measured the adsorption isotherm of CO_2 at 20 ºC with a desolvated sample of ZnW−**PYI**1 under high vacuum at room temperature. The uptake value of CO_2 was 1.36 cm^3g^{-1}(amount of gas at 20 ºC, 10 bar per gram of compound ZnW−**PYI**1, which corresponds to 0.06 mmol g^{-1}). Although the CO_2-adsorption capacity is far lower than that of some other recently reported MOFs, the material showed an excellent ideal activator of CO_2 gas. One reason for the low gas uptake is presumably the presence of POM and **PYI** groups in channels of ZnW−**PYI**1, which occupied a large portion of void space and thus decreased the accessible pore volume. From the irreversible CO_2-adsorption and desorption isotherms, we can deduced that the adsorption process mainly owed to the chemical adsorption.

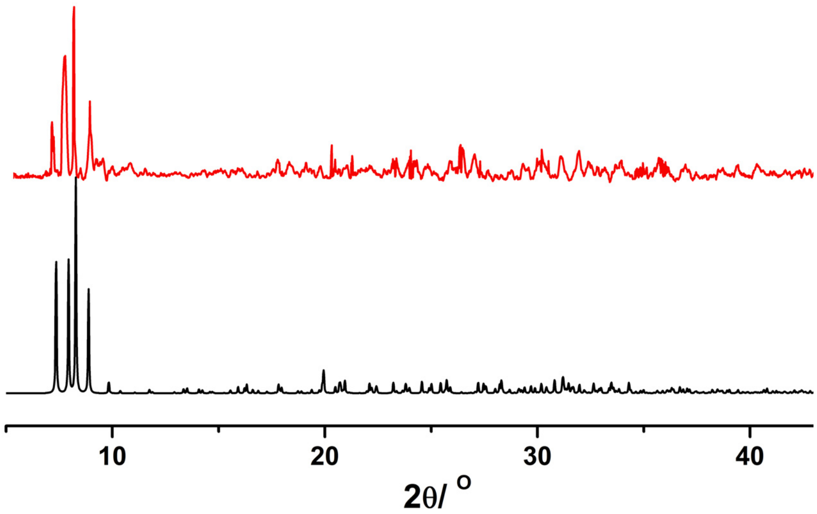

Supplementary Figure 14. PXRD pattern of ZnW−PYI2. Its calculated pattern based on the single-crystal solution and the Experimental patter (bottom-Simulated, top-Experimental).

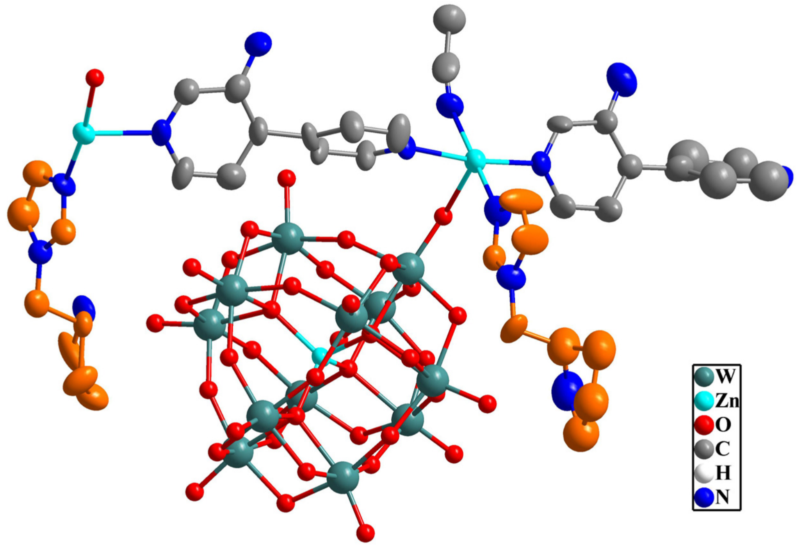

Supplementary Figure 15. Ellipsoid diagram of ZnW−PYI2 in an asymmetric unit with selected labelling scheme. All H atoms are omitted for clarity. The asymmetric unit of ZnW−**PYI**2 consists of the Keggin anion $ZnW_{12}O_{40}^{6-}$ and the cation $[Zn_2(NH_2$-**BPY**$)_2$(H**PYI**$)_2(H_2O)(CH_3CN)]^{6+}$ and one free CH_3CN molecule.

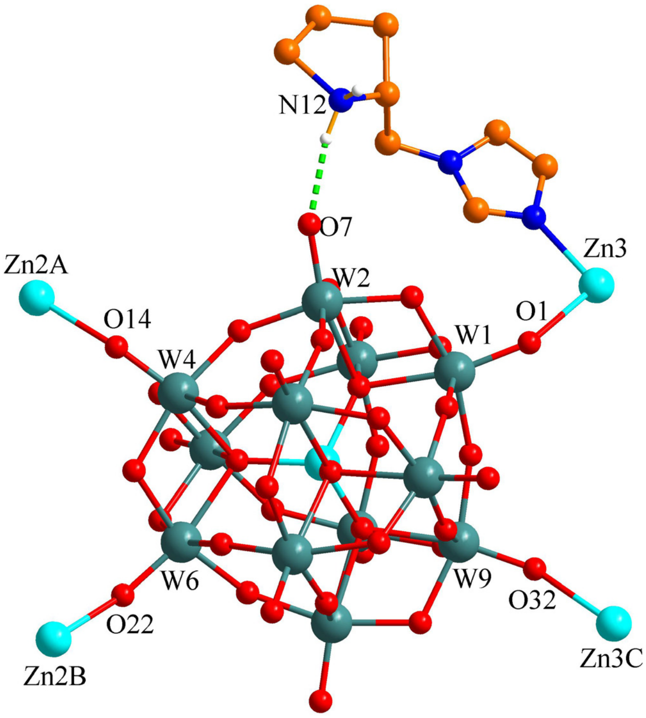

Supplementary Figure 16. Ball-and-stick representation of the coordination environments of $ZnW_{12}O_{40}^{6-}$ with selected labelling scheme, displaying hydrogen-bonding interactions between protonated pyrrolidine rings and the $ZnW_{12}O_{40}^{6-}$ in ZnW−**PYI**2. (Symmetry code**:** A 1+*x*, *y*, *z*; B 2-*x*, *y*+1/2, -*z*+1; C -*x*+2, *y*+1/2, -*z*+2)

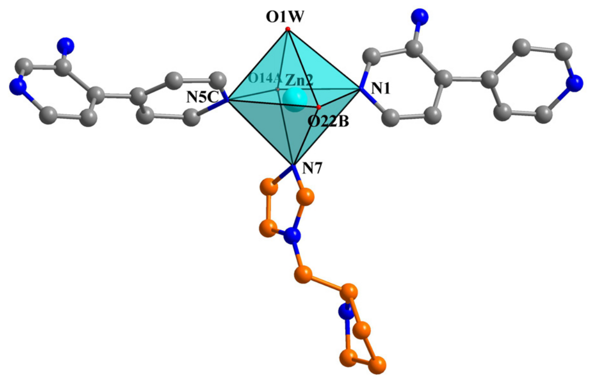

Supplementary Figure 17. Polyhedron of representation of the coordination environments of Zn(2) ion with selected labelling scheme in ZnW−**PYI**2 (Symmetry code**:** A *x*-1, *y*, *z*, B 2-*x*, *y*-1/2, -*z*+1, C *x*-1, *y*, *z*-1). The Zn(2) ion in ZnW−**PYI**2 adopts a distorted octahedral geometry. In $[Zn(2)(H_2O)(O_t)_2(N)_3]^{2+}$, Zn(2) atom is defined by one oxygen atom from coordinated water [Zn(2)–O(1W): 2.236(11) Å], two terminal oxygen atoms of $ZnW_{12}O_{40}{}^{6-}$ [Zn(2)–O(14A) 2.131(7) Å, Zn(2)–O(22B) 2.143(6) Å], two nitrogen atoms from NH_2-**BPY** [Zn(2)–N(1) 2.150(8) Å, Zn(2)–N(5C) 2.181(9) Å] and one nitrogen atom from **PYI** [Zn(2)–N(7) 2.057(8) Å].

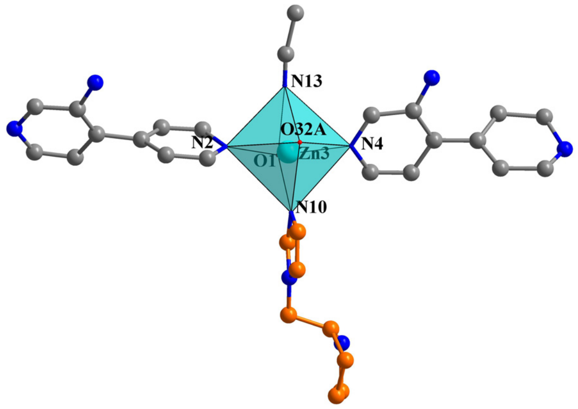

Supplementary Figure 18. Polyhedron of representation of the coordination environments of Zn(3) ion with selected labelling scheme in ZnW−**PYI**2 (Symmetry code**:** A -*x*+2, *y*-1/2, -*z*+2). The Zn(3) ion in ZnW−**PYI**2 adopts a distorted octahedral geometry. In $[Zn(3)(O_t)_2(N)_4]^{2+}$, Zn(3) atom is defined by two terminal oxygen atoms of $ZnW_{12}O_{40}{}^{6-}$ [Zn(3)–O(1) 2.273(6) Å, Zn(3)–O(32A) 2.200(6) Å], two nitrogen atoms from NH_2-**BPY** [Zn(3)–N(2) 2.133(7) Å, Zn(3)–N(4) 2.128(7) Å], one nitrogen atom from **PYI** [Zn(3)–N(10) 2.071(8) Å], and one nitrogen atom from CH_3CN [Zn(3)–N(13) 2.254(9) Å].

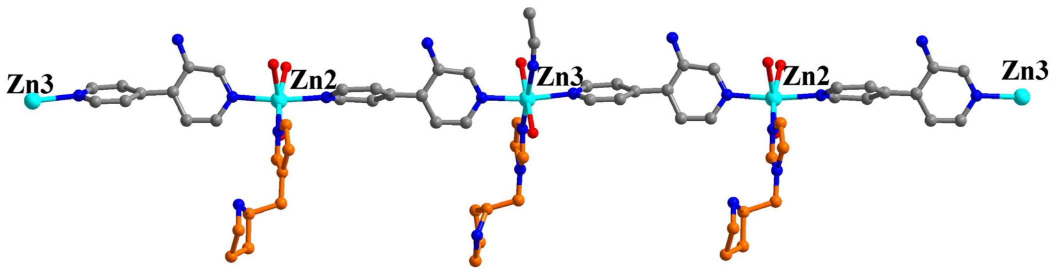

Supplementary Figure 19. The 1D chain of ZnW−**PYI**2 produced by two crystallographically independent Zn(II) ions linking with NH_2-BPY alternatively.

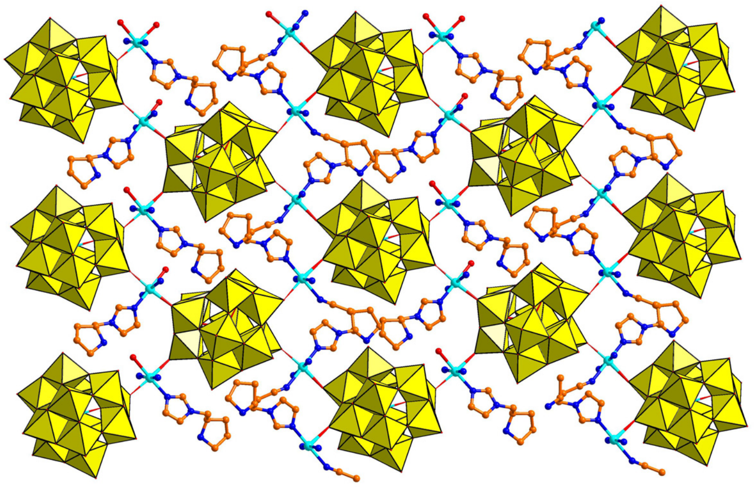

Supplementary Figure 20. The 2D network of ZnW−**PYI**2 showing the coordination environment of POM.

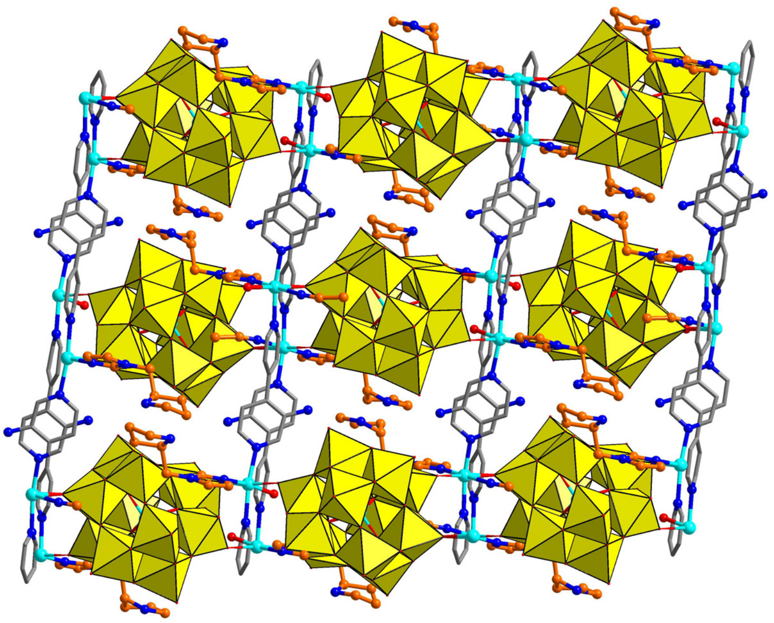

Supplementary Figure 21. Perspective view of the 3D open network of ZnW−PYI2 viewed down the *b*-axis. Carbon, nitrogen and zinc are drawn in gray (orange for PYI), blue, and cyan, respectively, with $ZnW_{12}O_{40}^{6-}$ shown as polyhedra. H-atoms and solvent are omitted for clarity.

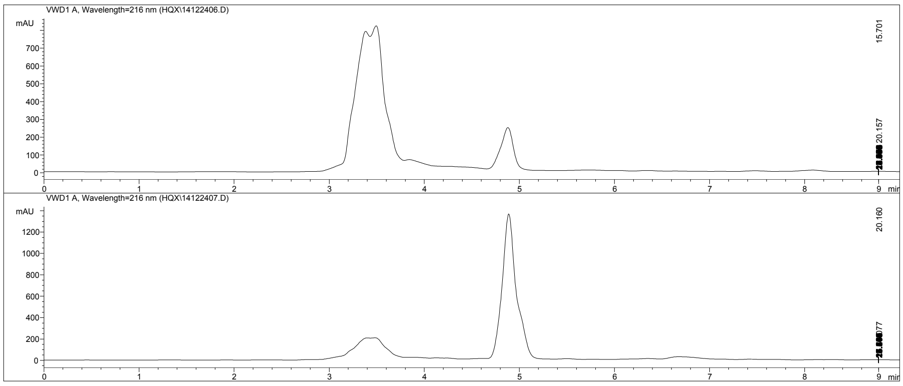

Supplementary Figure 22. HPLC spectra of (*R*)- (top) and (*S*)- styrene oxide (bottom).

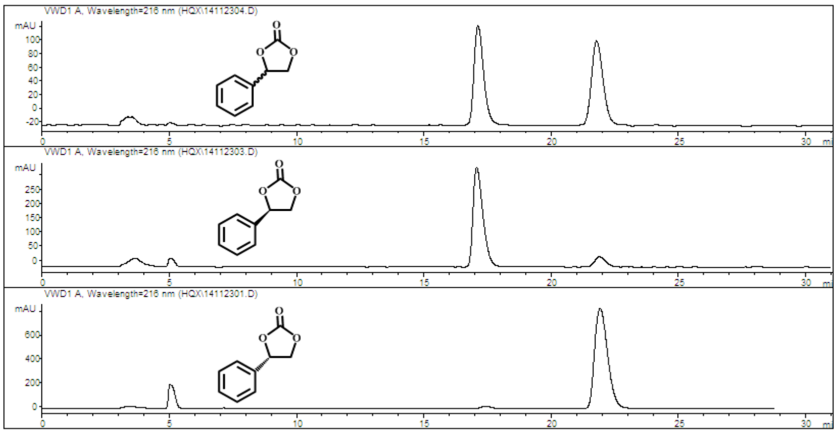

Supplementary Figure 23. HPLC spectra of racemic-, (*R*)- and (*S*)-phenyl(ethylene carbonate).

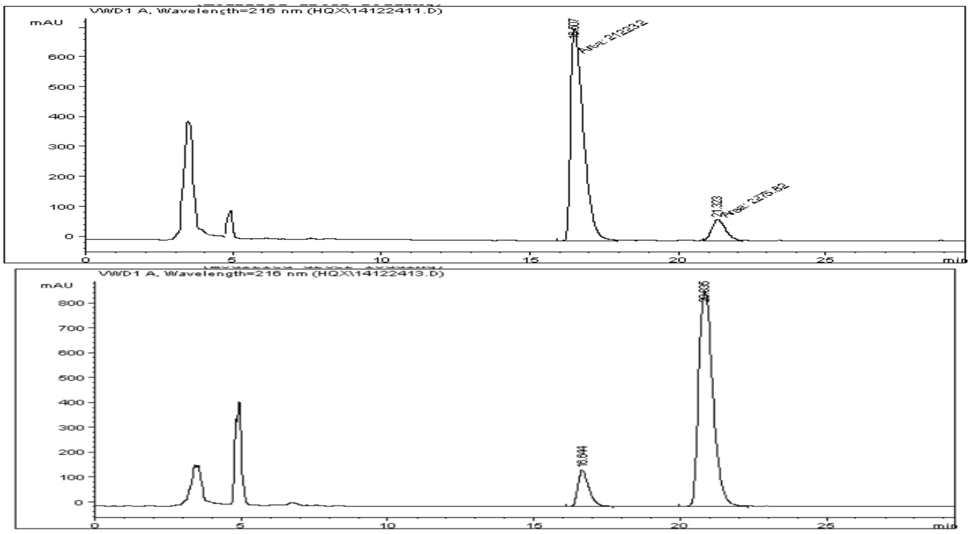

Supplementary Figure 24. HPLC spectra of racemic-, (*R*)- and (*S*)-phenyl(ethylene carbonate) by one-pot process. (Table 1, entry 9)

Supplementary Tables

Supplementary Table 1. Crystallographic data and structural refinements for ZnW−**PYI**1 and ZnW−**PYI**2.

	ZnW−**PYI**1	ZnW−**PYI**2
Empirical formula	$C_{40}H_{54}N_{14}O_{41}W_{12}Zn_3$	$C_{40}H_{54}N_{14}O_{41}W_{12}Zn_3$
M, g mol^{-1}	3789.28	3789.28
Crystal system	Monoclinic	Monoclinic
space group	$P2_1$	$P2_1$
a, Å	15.088(1)	15.103(1)
b, Å	13.235(1)	13.254(1)
c, Å	18.022(1)	18.033(1)
β, deg	94.41(1)	94.44(1)
V, Å^3	3588.2 (2)	3598.8(3)
Z	2	2
dcalcd, g cm^{-3}	3.507	3.497
T, K	296 (2)	296 (2)
Refl. Collected unique	19314/13673 R(int) = 0.0834	18434/13281 R(int) = 0.0699
μ, mm^{-1}	20.226	20.167
GOF	1.042	1.002
Flack parameter	-0.01(2)	-0.00(2)
F(000)	3396	3396
$R_1{}^a$ (I > 2σ(I)	0.0607	0.0608
$wR_2{}^b$ (I > 2σ(I)	0.1447	0.1417
$R_1{}^a$ (all data)	0.0662	0.0677
$wR_2{}^b$ (all data)	0.1479	0.1453
diff peak and hole, eÅ^{-3}	2.743/ -2.206	3.297/ -2.474

[a] $R_1 = \sum||Fo| - |Fc||/\sum|Fo|$, [b] $wR_2 = [\sum w(Fo^2 - Fc^2)^2/\sum w(Fo^2)^2]^{1/2}$; $w = 1/[\sigma^2(Fo^2) + (xP)^2 + yP]$, $P = (Fo^2 + 2Fc^2)/3$, where $x = 0.0753$, $y = 0$ for ZnW−**PYI**1; $x = 0.0701$, $y = 0$ for ZnW−**PYI2.**

Supplementary Table 2. Related bond Lengths (Å) of W=O_t in ZnW−**PYI**1.

Bond	Length(Å)	Bond	Length(Å)
W(2)-O(7)	1.745(8)	W(3)-O(11)	1.697(8)
W(5)-O(19)	1.733(8)	W(7)-O(25)	1.721(8)
W(10)-O(33)	1.727(8)	W(8)-O(39)	1.679(9)
W(1)-O(1)	1.758(8)	W(11)-O(36)	1.735(8)
W(4)-O(14)	1.738(10)	W(12)-O(40)	1.727(9)
W(6)-O(22)	1.726(7)		
W(9)-O(32)	1.758(7)		

Note: Average bond length for participating Hydrogen-bonding is 1.735(8) Å, for coordination with Zn(II) is 1.745(8) Å, and for nonparticipants is 1.712(8)Å, respectively. The average bond lengths of W=O_t participated in forming hydrogen bonding and coordination with Zn^{2+} are longer than that of nonparticipants, which was conducive to the activation of $ZnW_{12}O_{40}^{6-}$ to some extent.

Supplementary Table 3. The hydrogen bonds for ZnW−**PYI1**.

**D-H...A**	**d(H...A)**	**d(D...A)**	**<(DHA)**
N(3)-H(3A)...O(7)#1	2.24	3.101(17)	176.3
N(3)-H(3B)...O(33)#2	2.10	2.894(15)	152.9
N(6)-H(6A)...O(19)#3	2.44	3.086(17)	132.7
N(9)-H(38A)...O(26)	1.86	2.743(11)	167.2
N(9)-H(38B)...O(19)#4	2.08	2.867(13)	145.0
N(12)-H(12A)...O(7)	1.97	2.832(18)	159.4
N(12)-H(12B)...O(34)#5	2.04	2.934(18)	171.6

Symmetry transformations used to generate equivalent atoms: #1 *x*+1, *y*, *z*, #2 -*x*, *y*+1/2, -*z*, #3 *x*, *y*, *z*-1, #4 -*x*, *y*-1/2, -*z*+1, #5 -*x*-1, *y*+1/2, -*z*.

Supplementary Table 4. Related bond Lengths (Å) of W=O_t in ZnW−**PYI**2.

Bond	Length(Å)	Bond	Length(Å)
W(2)–O(7)	1.746(8)	W(3)–O(11)	1.607(8)
W(5)–O(19)	1.726(6)	W(7)–O(25)	1.709(7)
W(10)–O(33)	1.727(7)	W(8)–O(39)	1.717(6)
W(1)–O(1)	1.747(6)	W(11)–O(36)	1.746(7)
W(4)–O(14)	1.716(7)	W(12)–O(40)	1.708(7)
W(6)–O(22)	1.745(6)		
W(9)–O(32)	1.741(6)		

Note: Average bond length for participating Hydrogen-bonding is 1.733(7) Å, for coordination with Zn(II) is 1.737(6) Å, and for nonparticipants is 1.679(7)Å, respectively. The average bond lengths of W=O_t participated in forming hydrogen bonding and coordination with Zn^{2+} are longer than that of nonparticipants, which was conducive to the activation of $ZnW_{12}O_{40}^{6-}$ to some extent.

Supplementary Table 5. The hydrogen bonds for ZnW−**PYI2**.

D-H...A	d(H...A)	d(D...A)	<(DHA)
N(9)-H(38B)...O(26)	1.96	2.803(9)	154.9
N(9)-H(38B)...N(61)	2.55	3.016(17)	112.6
N(3)-H(3A)...O(33)#1	2.04	2.859(12)	158.4
N(3)-H(3B)...O(7)#2	2.29	3.127(12)	164.5
N(6)-H(6B)...O(19)#3	2.40	3.084(12)	136.7
N(12)-H(12B)...O(7)	1.96	2.819(15)	159.2
N(12)-H(12B)...O(34)#4	2.04	2.934(18)	171.6

Symmetry transformations used to generate equivalent atoms: #1 -*x*+2, *y*-1/2, -*z*+2 #2 *x*-1, *y*, *z* #3 *x*+1, *y*, *z* #4 *x*, *y*, *z*+1.

Supplementary Table 6. Peak results of (*R*)- and (*S*)- styrene oxide catalyzed by ZnW−**PYI**1 and ZnW−**PYI**2.

Cata.	Peak	RetTime [min]	Area mAU *s	Area %	ee %
ZnW−**PYI**1	1	3.436	389.29599	89.61	79.22
	2	4.926	88.20979	10.39	
ZnW−**PYI**2	1	3.481	102.44211	11.93	-76.14
	2	4.888	756.53140	88.07	

Supplementary Table 7. Influence of reaction parameters on the coupling of CO_2 to styrene oxide.

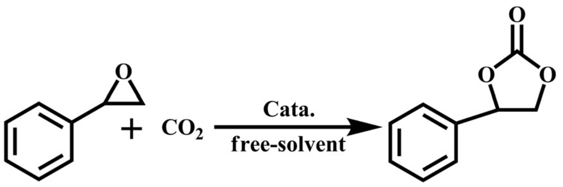

Entry	Substrate	Cata.	Co-cata.	Temperature	Time(h)	Yield(%)[b]	ee(%)[c]
s1	*R*-	ZnW−**PYI**1	TBABr	80	36	>99	75
s2	*R*-	ZnW−**PYI**1	TBABr	30	48	35%	97
s3	*R*-	ZnW−**PYI**1	TBABr	50	48	>99	90
s4	R,S-	ZnW−**PYI**1	TBABr	50	48	>99	trace
s5	R,S-	ZnW−**PYI**1	-	50	48	18	-
s6	R,S-	-	TBABr	50	48	13	-
s7	*S*-	ZnW−**PYI**2	TBABr	50	48	>99	-96

[a] Reaction conditions: styrene oxide: 5 mmol, catalyst: 0.005 mmmol, TBABr 0.05mmol, 0.5 MPa. [b]The conversions were determined by ^{1}H NMR spectroscopy of crude products. [c]The *e.e.*value was determined by chiral HPLC on a Chiralcel OD−H column.

Supplementary Table 8. Peak results catalyzed by ZnW−**PYI**1 and ZnW−**PYI**2.

Cata.	Styrene oxide	Peak	RetTime [min]	Area mAU *s	Area %	ee %
ZnW−**PYI1**	Racemic	1	17.127	146.69	49.46	–
		2	21.786	124.81	50.54	
ZnW−**PYI**1	R-	1	17.079	351.93	95.00	90.00
		2	21.905	18.53	5.00	
ZnW−**PYI**2	S-	1	17.423	22.35	2.01	-95.98
		2	21.927	7518.59	97.99	

Supplementary Table 9. Peak results catalyzed by ZnW−**PYI**1 and ZnW−**PYI**2 in one-pot process.

Cata.	Peak	RetTime [min]	Area mAU *s	Area %	ee %
ZnW−**PYI**1	1	16.507	2.12232 e4	90.32	80.00
	2	21.323	2275.81909	9.68	
ZnW−**PYI**2	1	16.644	146.75	11.45	-77.11
	2	20.835	863.57	88.55	

Supplementary Table 10. Study on the recycling of catalyst ZnW−**PYI**1 for the heterogeneous auto-tandem catalysis of styrene.

**Entry**	**Yield(%)**	**ee(%)**
Round 1	92	80
Round 2	91	78
Round 3	88	77

Supplementary Methods

Typical procedure for asymmetric epoxidation of aromatic olefin using chiral POMOFs. The catalytic epoxidation reactions of styrene by TBHP carried out heterogeneously in 10 mmol styrene, 0.01 mmmol catalyst, 20 mmol TBHP without extra solvent under stirring in a round bottom flask fitted with a water condenser and placed in an oil bath at 50 °C for 120h. After completion of the reaction, the mixture was filtered and extracted. The organic extracts were dried over Na_2SO_4 and evaporated to dryness. The yields were directly determined by ^{1}H NMR analysis of the reaction solution. Absolute configurations were determined by chiral HPLC on a Chiralcel OD−H column.

^{1}H NMR analysis and chiral HPLC analysis of (*R*)-styrene oxide.

(*R*)-styrene oxide: ^{1}H NMR (400 MHz, $CDCl_3$): δ = 7.32–7.36 (m, 5H), 3.83–3.85 (m, 1H), 3.11–3.14(m, 1H), 2.77–2.79 (m, 1H). Enantiomeric excess was determined by HPLC with a Chiralcel OD−H (250 mm × 4.6 mm) columns; UV detection at 216 nm; a mixture of *n*-hexane and 2-propanol (85:15) was used as mobile phase with a flow of 1.0 mL/min, 45 bar. The following figures are the chiral HPLC chromatograms of (*R*)- and (*S*)- styrene oxide prepared. The retention times of (*R*)-

and (*S*)- phenyl(ethylene carbonate) are about 3.4 min and 4.8 min, respectively. (see Supplementary Figure 22 and Supplementary Table 6)

(*R*)-4-methylstyrene epoxide: ^{1}H NMR (400 MHz, $CDCl_3$): δ = 7.34–7.37 (m, 4H), 3.84–3.86 (m, 1H), 3.12–3.15(m, 1H), 2.77–2.80 (m, 1H), 2.36-2.38 (m, 3H). Enantiomeric excess was determined by HPLC with a Chiralcel OD−H (250 mm × 4.6 mm) columns; UV detection at 216 nm; a mixture of *n*-hexane and 2-propanol (95: 5) was used as mobile phase with a flow of 1.0 mL/min, 45 bar. The retention times of (*R*)- and (*S*)- phenyl(ethylene carbonate) are about 7.5 min and 9.8 min, respectively.

(*R*)-4-tert-butylstyrene epoxide: ^{1}H NMR (400 MHz, $CDCl_3$): δ = 7.46–7.49 (m, 4H), 3.84–3.86 (m, 1H), 3.132–3.16(m, 1H), 2.78–2.82 (m, 1H), 1.36-1.38 (m, 9H). Enantiomeric excess was determined by HPLC with a Chiralcel OD−H (250 mm × 4.6 mm) columns; UV detection at 216 nm; a mixture of *n*-hexane and 2-propanol (95: 5) was used as mobile phase with a flow of 1.0 mL/min, 45 bar. The retention times of (*R*)- and (*S*)- phenyl(ethylene carbonate) are about 9.4 min and 11.8 min, respectively.

(2*S*,3*R*)-3-Phenyl-oxirane-2-carbaldehyde: ^{1}H NMR (400 MHz, $CDCl_3$): δ = 9.21(s, 1H) 7.22–7.36 (m, 5H), 4.13–4.15 (m, 1H), 3.39–3.41(m, 1H). Enantiomeric excess was determined by HPLC with a Chiralcel OD−H (250 mm × 4.6 mm) columns; UV detection at 216 nm; a mixture of *n*-hexane and 2-propanol (95: 5) was used as mobile phase with a flow of 1.0 mL/min, 45 bar. The crude reaction mixture, from the epoxidation of *α, β*-unsaturated aldehydes described above, was diluted with MeOH (1.0 mL) and cooled to 0 °C followed by addition of $NaBH_4$ (19 mg, 0.5 mmol). The mixture was stirred for 10 mins, quenched with sat. NH_4Cl and extracted with Et_2O. Then the ee of the resulting epoxide was determined by chiral GC analysis of the diol derivative from hydrolysis with 1M NaOH. The retention times of 2,3-dihydroxy-3-phenylpropanal enantiomers are about 19 min and 23 min, respectively.

Typical procedure for the coupling reaction of CO_2 with epoxides. The reactions of CO_2 and epoxides were carried out in a 50 mL stainless steel autoclave equipped with a magnetic stirrer. In a typical procedure, catalyst (0.01 mmol), TBABr (0.1 mmol) and styrene oxide (10 mmol) was added to a Schlenk flask (50 mL) equipped with a three-way stopcock. Then CO_2 was charged into the autoclave, and the pressure (0.5 MPa) was kept constant during the reaction. The autoclave was put into a bath

and heated to the desired temperature. After the expiration of the desired time, the excess gases were vented. The remaining mixture was degassed and fractionally distilled under reduced pressure or purified by column chromatography on silica gel to obtain the cyclic carbonate. Enantiomeric excesses of cyclic carbonates derived from styrene oxide were determined by chiral HPLC on a Chiralcel OD-H column (Daicel Inc., 25 cm × 0.46 cm i.d.).

The experiments shown in Table S7 were conducted to examine the influence of temperature and reaction time. The transformation of the asymmetric coupling of CO_2 to styrene oxide was examined initially by using the (*R*)-styrene oxide and CO_2 in free-solvent, along with ZnW−**PYI**1(0.1% mol ratio) with co-catalyst TBABr (1% mol ratio) in a heterogeneous manner at 80 ℃ and 0.5 MPa for 36 h, as shown in Table 1. The result revealed the successful execution of our MOF design, showing the excellent reaction efficiency (>99% in *yield*) and enantioselectivity (75% in *ee*) for (*R*)-phenyl(ethylene carbonate) (entry s1). When the reaction temperature decreased from 80 ℃ to 30 ℃ led to dramatic increases in enantiopurity of the formed phenyl(ethylene carbonate) from 75 to 97% *ee* (entry s2), although the reaction efficiency was decreased from 100% to 35%. The highest yield of 99% and highest *ee* of phenyl(ethylene carbonate) was obtained under optimal conditions, that is at 50℃ and reaction temperature 48 h (entry s3). As the phenyl (ethylene carbonate) obtained through the coupling of CO_2 to the racemic styrene oxide did not exhibit any enantioselectivity, the enantioselectivity of the coupling thus majorly attributes to the asymmetric behavior of the enantiopure epoxide substrate (entry S4). It is worthwhile to note here that the CO_2/styrene oxide coupling catalyzed by ZnW−**PYI**1 in conjunction with an ammonium salt, a high yield was observed, however, when only in the present of ZnW−**PYI**1 or co-catalyst TBABr, the reaction was inefficient (entries S5 and S6). In addition, the removal of ZnW−**PYI**1 by filtration after 24 hours shut down the reaction, and the filtrate afforded scarcely additional conversion after stirring at 80 ℃ for another 24 hours. These observations suggested that ZnW−**PYI**1 was a true heterogeneous catalyst. Solids of ZnW−**PYI**1 could be isolated from the reaction suspension by simple filtration alone. And the catalysts could be reused at least three times with moderated loss of activity (from > 99% to 97% of yield) and slight decrease in the selectivity (from 90% to 89% of *ee*). Solids of ZnW−**PYI**2 exhibited similar catalytic activities but gave products with opposite

chirality in the asymmetric coupling reaction of CO_2 with (*S*)-styrene oxide (entry s7).

¹H and ¹³C NMR analysis and chiral HPLC analysis of cyclic carbonates.

(*R*)-phenyl(ethylene carbonate): ^{1}H NMR (400 MHz, $CDCl_3$): δ = 7.36–7.45 (dd, 5H), 5.66−5.70 (t, 1H), 4.78−4.82(t, 1H), 4.33−4.37 (t, 1H). ^{13}C NMR (126 MHz, $CDCl_3$):δ 154.82, 135.66, 129.74, 129.25, 125.87, 77.99, 71.17.[1]

Chiral HPLC analysis: Enantiomeric excess was determined by HPLC with a Chiralcel OD−H (250 mm × 4.6 mm) columns; UV detection at 216 nm; a mixture of *n*-hexane and 2-propanol (85:15) was used as mobile phase with a flow of 1.0 mL/min, 45 bar. The following figures are the chiral HPLC chromatograms of (*R*)- and (*S*)- cyclic carbonates prepared. The retention times of (*R*)- and (*S*)- phenyl(ethylene carbonate) are about 17 min and 21 min, respectively. (Table 1, entry 5-7, see Supplementary Figure 23 and Supplementary Table 8)

Typical one-pot procedure for the transformation of olefins to chiral cyclic carbonates using chiral POMOFs.

Catalyst (0.01 mmol), TBABr (0.1 mmol), TBHP (20 mmol) and styrene (10 mmol) was added to a Schlenk flask (50 mL) equipped with a three-way stopcock. Then CO_2 was charged into the autoclave, and the pressure (0.5 MPa) was kept constant during the reaction. The autoclave was put into a bath and heated to the 50 °C. After the expiration of the desired time, the excess gases were vented. The remaining mixture was degassed and fractionally distilled under reduced pressure or purified by column chromatography on silica gel to obtain the cyclic carbonate. (see Supplementary Figure 24 and Supplementary Table 9)

Typical one-pot procedure for the transformation of propylene to cyclic carbonate using chiral POMOFs.

Catalyst (0.01 mmol), TBABr (0.1 mmol), TBHP (20 mmol) and propylene (12 mmol, 0.5 MPa) was added to a Schlenk flask (50 mL) equipped with a three-way stopcock. Then CO_2 was charged into the autoclave, and the pressure (1.0 MPa) was kept constant during the reaction. The autoclave was put into a bath and heated to the 50 °C. After the expiration of the desired time, the reactor was put into ice bath for 10 min and depressurized, and a small aliquot was taken to be analyzed by ^{1}H NMR and ^{13}C NMR using mesitylene as the internal standard to calculate the conversion of propylene oxide.

Propylene carbonate: ^{1}H NMR (400 MHz, $CDCl_3$): δ = 5.06–4.98 (m, 1H), 4.59 (t, 1H), 4.04(t, 1H), 1.50–1.45 (t, 3H). ^{13}C NMR (126 MHz, $CDCl_3$): δ 154.8, 73.9, 19.8.[2]

Supplementary References

1. Wang, J. L. et al. A CO_2/H_2O_2-tunable reaction: direct conversion of styrene into styrene carbonate catalyzed by sodium phosphotungstate/n-Bu_4NBr, *Green Chem.* **10**, 1218–1223 (2008).

2. Gao, W. Y. et al. Crystal engineering of an *nbo* topology metal–organic framework for chemical fixation of CO_2 under ambient conditions. *Angew. Chem. Int. Ed.* **53**, 2615–2619 (2014).
